# Multiple sclerosis projection in Tehran, Iran using Bayesian structural time series

**DOI:** 10.1186/s12883-021-02281-x

**Published:** 2021-06-24

**Authors:** Payam Amini, Amir Almasi-Hashiani, Mohammad Ali Sahraian, Masood Najafi, Sharareh Eskandarieh

**Affiliations:** 1 Department of Biostatistics and Epidemiology, School of Public Health, Ahvaz Jundishapur University of Medical Sciences, Ahvaz, Iran; 2 Department of Epidemiology, School of Health, Arak University of Medical Sciences, Arak, Iran; 3 Multiple Sclerosis Research Center, Neuroscience Institute, Tehran University of Medical Sciences, Tehran, Iran

**Keywords:** Multiple Sclerosis, Prevalence, Time series, Tehran

## Abstract

**Background:**

The prevalence of Multiple Sclerosis (MS) has been increasing worldwide and the highest prevalence ratio among Asian countries was reported in Iran. This study aims to estimate the increase in MS occurrence during more than three decades in Tehran and forecast the future condition of the disease using time series approaches for the next ten years.

**Methods:**

The cross-sectional study was conducted from 1999 to 2019 based on records of MS cases from Iranian MS Society (IMSS) registry system. The prevalence was estimated using population data presented by the Statistical Centre of Iran. Through Bayesian Structural Time Series (BSTS) model, we want to predict the prevalence of familial and sporadic MS in the next ten years. .

**Results:**

Among 22,421 cases with MS, 16,831 (75.1 %) were female and 5589 (24.9 %) were male. Female to male ratio was 3.0:1 and the number of familial MS cases were 2982 (13.3 %) of subjects. Female gender was less responsible for higher rate of MS in familial definition (beta = 0.020) in comparison to sporadic cases (beta = 0.034). Forecasting by BSTS revealed an increase in MS prevalence for the next ten years so that the prevalence rate for total, familial and sporadic MS respectively begins with 189.50 (183.94-195.14), 25.69 (24.97–26.45) and 163.74(159.06-168.57) in 2020 and ends with 220.84 (171.48-266.92), 30.79 (24.16–37.15), and 189.33(146.97-230.19) in 2029.

**Conclusions:**

According to the findings, MS prevalence increased during three decades and it will increase over the next ten years. Tehran province is one of the regions with highest MS prevalence in Asia. The results of present study indicated that females are at higher risk for MS than males in both sporadic and familial MS.

## Introduction

Multiple sclerosis (MS) is a neurological condition that impacts the central nervous system and is known as the most common non-traumatic disabling disease [1]. The immune system attacks the protective layer around nerve fibers and destroys the myelin and the axons in different grades [2]. MS has a highly complex and inconsistent course. In majority of patients, the disorder starts with reversible neurological deficits, which are often accompanied by gradual neurological decline over time [3]. Although weakness and numbness are typical symptoms of this progressive and immune-mediated disorder, extreme cases may result in paralysis, vision loss, and cognition problems [4].

Traditionally, MS was considered to be an organ-specific T-cell associated autoimmune disorder. The effectiveness of B-cell targeted therapies, on the other hand, brings into doubt the traditional T-cell autoimmune theory [5]. Multiple external factors and genetic alleles can affect the likelihood of experiencing multiple sclerosis, but the disease’s underlying cause remains uncertain [6]. MS patients have a broad variety of symptoms which differ greatly between individuals due to the severity of the condition [7]. Debilitation and fatigue are the most common symptoms of MS and can be followed by numbness in muscles, sensory impairment, spasticity, pain, depression, difficulties in balancing and vision, acute or chronic pain, tremor, and cognitive problems in concentration and memory [8, 9]. Although many medications and interventions have been introduced to prevent the progression of neurological disability, many cases still meet the long-term course of multiple sclerosis [10].

Many studies have been performed on the epidemiology of multiple sclerosis. Prevalence has grown gradually in most of developed and developing countries over the last five decades, especially with high concentrations among women [11]. Based on the studies compiled by Multiple Sclerosis International Federation (MSIF), the Atlas of MS (www.atlasofms.org), the estimated number of people with MS worldwide has reached to 2.8 million in 2020 which is almost 30 % higher than that of 2013. The research has reported the 2020 global prevalence of 35.9 [95 % CI: 35.87, 35.95] per 100,000 population [11]. According to several studies, MS is becoming more prevalent in the Middle East and North Africa [12, 13]. These regions have low-to-moderate incidences of MS, with rates lower than Southern Europe but considerably higher than Sub-Saharan Africa. Iran has always had the highest MS incidence in the region, rising from 51.9 to 2010 to 148.06 per 100,000 population in 2017, likely due to genetic factors linked to the Iranian population’s multiple ethnic backgrounds [14–16]. Early diagnosis, particularly with the advent of magnetic resonance imaging in the 1980 s and the new McDonald diagnostic criteria, may be part of the explanation for the rapid increase in prevalence rates in the Middle East region [17].

MS is related to higher levels of depression and anxiety, poorer quality of life, inefficient personal relationships, high economic costs to society, and high levels of healthcare utilization [18]. Thus, it is very important to investigate the trend of MS during the decades and to predict the future status of the disease to reduce the adverse consequences of MS on the individual, families and society. This study aims to evaluate the development of MS in Tehran during more than three decades and forecast the future condition of the disease for the next ten years using time series approaches.

## Methods

### Study area

This population-based study was conducted in Tehran Province based on the Iranian MS Society (IMSS) registry system. Tehran is capital of Iran located in the northern central of the country. (Latitude: 35 ° North, Longitude: 51° East).

### Data source and participants

Iranian MS Society (IMSS) records were considered to obtain annual incidence data from 1st April 1999 to 31st December 2019 [13, 15]. The IMSS is the only center in Tehran that registers MS patient data and provides wide services such as rehabilitation and social health facilities, mental and medical services for the members. Only patients residing in Tehran area who have been approved by neurologists and diagnosed based on McDonald criteria are registered in the IMSS.

### Study design

To design the cross-sectional population based study, the questionnaire designed in MS research center of Tehran University of medical sciences covered the important epidemiological variables associated to risk for recurrence of MS including sex, age at disease onset and familial history of MS. A trained interviewer explains the aims of the registry for participants in the IMSS, and after taking informed consent, patients are asked to complete structured questions [19].

The MS prevalence estimate was calculated by the population data achieved from the Statistical Centre of Iran.

The Statistical Center of Iran regularly conducts population censuses, including in the year 2020, and estimates the average annual population for the in-between years and accordingly, it predicts the population of coming years based on several registries available in the country.

The questionnaire is designed to determine the prevalence of MS and to identify the demographic characteristics and patients’ needs because the MS association is a center that provides a wide range of services, including social, financial and medical support to the patients. All patients are interviewed daily in the MS association for registration.

### Patients’ classification

Patients were categorized as familial MS (FMS) and sporadic MS (SMS) groups. The patients who had at least one affected relative were assigned to the FMS group, while subjects who were the only family member with MS were assigned to the SMS group [16].

### Ethical consideration

This study was approved by institutional review board of Tehran University of Medical Sciences, Ethical approval to perform this study was obtained by code (IR.TUMS.NI.REC.1399.032). All methods were performed in accordance with the relevant guidelines and regulations.

### Statistical analysis

The descriptive statistics of data was reported as mean (standard deviation) or frequency (percentage).

#### Bayesian Structural Time Series model (BSTS)

The BSTS approach is characterized by two equations. Firstly, the covariates and factors ($X$) affect the series via the coefficients ($\beta$) and the latent variables ($\alpha$) are related to the stochastic process by structural parameters ($Z$). The error terms $\epsilon$ and $\tau$ follow independent normal distributions. The latent variable evolves through time using the second equation.

$${Y}_{t}={\beta X}_{t}+{Z}_{t}{\alpha }_{t}+{\epsilon }_{t}$$

$${\alpha }_{t+1}={T}_{t}{\alpha }_{t}+{\tau }_{t}$$

The latent or unobserved trend ($\alpha$) represents an underlying change in the development of MS that is hard to pinpoint and should be controlled with explicit terms. The estimation procedure is carried out at the same time which avoids inflation type I error and strange coefficient estimates. The Bayesian nature of the method improves the estimations regarding the short series of prevalence rates over the years. As well as the estimated coefficients, the inclusion probability of each independent variable is visualized to find the variables which the model is dominated by. More details about the approach can be found elsewhere [20–23].

Data analyses were done using “bsts” package in R programming software version 4.0.4 (https://www.R-project.org/).

## Results

**Fig. 1:**
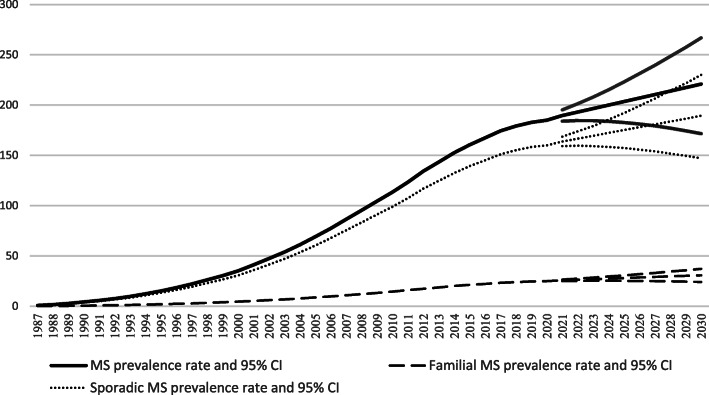
The prevalence of MS from 1987 to 2019 and forecasting tor the next 10 years (prevalence in 100,000 population)

Among 22,421 cases with MS, 5589 (24.9 %) were male and 16,831 (75.1 %) were female. Among MS patients, 18,805 (83.9 %) were sporadic, 2982 (13.3 %) were familial and 634 (2.8 %) were unknown. The frequency (percentage) of males and females in sporadic data was 4389 (24.4 %) and 13,601 (75.6 %) respectively. The mean (standard deviation) age of patients in the total, sporadic and familial data was 28.95 (3.01), 29.14 (3.35), and 27.63 (2.47). Regarding the development of MS prevalence (in 100,000 population) shown in Fig. 1, the minimum prevalence rate was observed in 1987 (total data: 0.78, Familial: 0.10, and sporadic: 0.68 per 100,000 population). After an almost constant rate, the increasing trend starts in 1998 (total data: 26.32, Familial: 3.43, and sporadic: 22.9 per 100,000 population) and gets accelerated in 2008 (total data: 95.44, Familial: 12.02, and sporadic: 83.42 per 100,000 population). The maximum prevalence rate is observed in 2019, 1998 (total data: 184.88, Familial: 24.99, and sporadic: 159.89 per 100,000 population).

The results of BSTS model for evaluating the prevalence rate of MS using covariates are shown in Table 1. One percent increase in female population, increases the prevalence rate of MS by 0.25. Older age is associated with 0.01 higher prevalence and sporadic MS rises the rate by 1.9 per 100,000 populations. Female gender was less responsible for higher rate of MS in familial (beta = 0.020) cases in comparison to sporadic (beta = 0.034).

**Table 1:** The results of BSTS model for assessing the development of MS

**Data**	**Variable**	**Beta**	**SE**	***P*-value**
All	Female	0.249	0.029	<0.001
	Age	0.011	0.001	<0.001
	Familial	-1.919	0.110	<0.001
Sporadic	Female	0.034	0.007	<0.001
	Age	0.009	0.007	0.205
Familial	Female	0.020	0.001	<0.001
	Age	0.009	0.002	<0.001

The BSTS model was used for forecasting and the prevalence rate (95 % confidence interval) from 2020 to 2029 are reported in Table 2. It should be mentioned that the point forecast estimates for more distant times might be discussed with more cautious regarding their wider confidence intervals. Using the information from Table 2; Fig. 1, a monotone increase in MS prevalence is predicted for the next ten years after 2020. The prevalence rate for MS in the total, familial and sporadic sets respectively begins with 189.50(183.94-195.14), 25.69(24.97–26.45), and 163.74(159.06-168.57) in 2020 and ends with 220.84(171.48-266.92), 30.79(24.16–37.15), and 189.33(146.97-230.19) in 2029.

**Table 2:** Forecasting the prevalence (95 % confidence interval) of MS from 2020 to 2029 (prevalence in 100,000 population)

Year	Total MS	Familial MS	Sporadic MS
2020	189.50(183.94-195.14)	25.69(24.97–26.45)	163.74(159.06-168.57)
2021	193.00(184.6-201.28)	26.26(25.18–27.39)	166.6(159.61-173.82)
2022	196.50(184.42-208.02)	26.83(25.23–28.35)	169.45(159.15-179.16)
2023	199.97(183.79-215.27)	27.4(25.24–29.49)	172.32(158.38-185.68)
2024	203.45(182.7-222.98)	27.96(25.17–30.61)	175.11(157.29-192.05)
2025	206.93(181.19-231.26)	28.53(25.1-31.83)	177.96(155.73-199.19)
2026	210.41(179.31-239.54)	29.09(24.92–33.11)	180.82(154.02–206.5)
2027	213.87(176.92-248.41)	29.66(24.72–34.46)	183.63(151.76-214.43)
2028	217.36(174.36–257.50)	30.23(24.47–35.72)	186.51(149.58-221.73)
2029	220.84(171.48-266.92)	30.79(24.16–37.15)	189.33(146.97-230.19)

## Discussion

The present research investigated the prevalence of MS in Tehran, Iran’s capital, from 1987 to 2019. According to the findings, MS is becoming more prevalent in Tehran, with the prevalence increasing from 0.78 cases per 100,000 people in 1987 to 184.88 in 2019. Moreover, the sporadic and familial MS prevalence rate increases from 0.68 to 159.89 and 0.10 to 24.99 per 100,000 populations. We showed that females are in a higher danger of MS than males in both sporadic and familial MS based on the total MS data. .We also demonstrated that the older age is significantly associated with higher prevalence of MS.

Iran is introduced as a country with a high number of MS cases, not only in the Middle East region, but also in Asia and the world [11, 24]. Among several provinces in Iran, Tehran has the highest prevalence rate of MS [25]. It has been well discussed that ethnicity might be responsible for MS occurrence so that MS is more prevalent in some ethnics [26]. Different ethnics are located in Iran such as Lor, Kurd, Pars, Turk and etc. and that might explain the high prevalence of MS. Abdollahpour et al. assessed the association between parental ethnicity and multiple sclerosis using a population based study in Iran and they exposed that Lor ethnicity was significantly associated with higher MS onset [26]. In a survey in the United Kingdom by Christo Albor, substantial differences in MS prevalence were identified in distinct racial clusters, namely the White, Black, and South Asian populations [27]. Although the majority of the residents in the capital city of Iran are mostly Pars, people from different ethnics live in Tehran due to different reasons such as lack of balanced economic development, inequality in access to facilities, and etc. Thus ethnicities are unequally distributed in the capital and the occurrence of MS might be more complex to study in Tehran. It is essential to know that Iranians heredity and genetic structure differ from other countries in the Middle East region. It has been argued that the occurrence of MS in Tehran is not the same as other provinces in Iran. Based on previous population based studies on geographic variations of MS in Iran, the high MS rate in Tehran is connected to several causes such as immigrations from rural regions and small cities to the capital, social and economic status, and air pollution. Besides, access to medical care in Tehran is significantly easier than other cities in Iran which justifies the higher prevalence rate due to routine diagnosis and case registration [28].

The Bayesian model showed that females are and will be responsible for higher prevalence of MS. Multiple reasons have been introduced that women have an earlier onset of MS and are less associated with lower prevalence of primary progressive disease [29]. It has been widely studied that women are at a higher risk of MS due to genetics. Exposure to sun and the level of Vitamin D have been suggested as the potential factors for MS. External conditions such as sun exposure and vitamin D supplementation can have various effects on men and women. According to this, higher vitamin-D levels have been related to a lower risk of MS, especially in women, due to biological variations [30]. Knudsen et al. reported that women’s X chromosome inactivation may be skewed, leading to an overrepresentation of MS susceptible genes in the female population [31]. In addition, studies have postulated that TLR7, CD40L, and FoxP3 are the potential genes on the X chromosome that cause different expressions which might attribute to the X dosage effect [32]. In addition to genetic and environmental factors, it is known that pregnancy suppresses the mother’s immune system to keep the fetus from being rejected, and therapies used during assisted reproductive techniques may also affect the development of MS by altering the patient’s hormonal status [33]. Researchers have shown that the rate of infertility is growing worldwide and to prevent infertility related psychological and other adverse consequences, the rate of assisted reproductive techniques have been raised as well [34, 35].

We also showed that sporadic MS in Tehran significantly increases the MS prevalence rate. For sporadic cases, environmental causes is more significant, while genetic vulnerability is more important for familial MS [36]. Genetic variants are found in patients with familial MS more than sporadic cases. The other main difference between the sporadic and familial MS is the time of diagnosis from the disease onset. Moreover and when opposed to intermittent MS cases, familial MS cases are diagnosed at a younger age [36]. Tipirneni et al. investigated the Magnetic Resonance Imaging (MRI) characteristics of familial and sporadic MS patients. They demonstrated that familial MS is correlated with more severe T1-lesion volume and its MTR characteristics. Moreover, no clinical differences were found between the two groups of MS patients [37]. Growing number of new MS cases among family members in future has been reported and there are variations in the clinical path and survival between the genders of intermittent and familial cases [38]. Distribution of MS courses in familial and sporadic cases was assessed by Steenhof et al. They found that in contrast to sporadic MS cases, familial MS patients are more prone to have relapsing-remitting MS than progressive MS. Moreover, first-degree relatives in MS families are more likely to have the same MS path [39]. Another study by Rojas et al. was conducted to evaluate the MS disorder among two groups of patients. It was observed that older generations are almost the same regarding the age at onset. Moreover, a noteworthy lower age at onset in the younger groups of familial MS cases was found compared to that sporadic [40].

The Bayesian model forecasted the MS prevalence rate for the next ten years and demonstrated an increasing trend. The increasing trend and forecasting have been reported by the study of Mousavizadeh et al. in which an increasing development of MS incidence was observed up to 2024 [41]. In addition to the ethnic, genetic and environmental factors, another potential reason for the increase in future might be related to the fact that the social awareness and information about the disease have been promoted during the recent years and individuals with the primary and mild symptoms might refer to physicians for early diagnosis. The MS cases are usually introduced as certain patients who are under an adequate and perfect insurance coverage in Iran and this leads to more recognition of new cases [41]. Tehran has been dealing with drought, increase in average annual temperature, dust, and air pollution during the recent decades and the condition is prone to get worse [42]. Moreover, Iran has experienced eight years’ war with the western neighbor, Iraq, during 1981 and 1989 and this is a significant environmental factor for the increase in the disease. The war disasters are responsible for negative psychological consequences after 1989 where the increase in the MS prevalence rate begins in Tehran [41]. The increasing trend of MS is strongly associated with the increasing advances in brain and magnetic resonance imaging which significantly supports the clinicians in diagnosing MS and helps to detect different components of MS pathogenesis {Cortese, 2019 #150}. It is important to study the diagrams forecasting the disease pattern for the next few years, in order to build strategies and acquire appropriate coping preparations. We forecasted a new, substantial outbreak of MS in the next decade, considering the recent growth pattern in disease exploration. If the appropriate infrastructures for tracking and handling these patients are not considered, the future wave will cause a variety of adverse consequences. It’s worth mentioning that, owing to the disease’s low mortality rate, patients live for a long time and this will raise the disease’s cumulative prevalence rate during years.

### Limitation

The numbers of patients and prevalence ratio may be underestimated, because some of cases might not have been registered during the years of disease onset. It should be highlighted that this study used population-based data, but it is focused on a single center (Tehran province). Therefore, the results of this study may not be generalizable to the whole country or other regions around the world, and it is recommended that the results be generalized with caution.

## Conclusions

The disease’s prevalence in the current study is in line with regional and global trends. The forecasting approach used to analyze the data indicates an important increasing trend in MS prevalence rate during the next ten years, which can be a key subject for additional research by researchers.

## Data Availability

The datasets used and/or analyzed during the current study available from the corresponding author on reasonable request.
